# Trends in dengue incidence and lethality: interrupted time series analysis, Brazil, 2001-2022

**DOI:** 10.1590/S2237-96222025v34e20240424.en

**Published:** 2025-09-08

**Authors:** Tatiane Fernandes Portal de Lima Alves da Silva, Henry Maia Peixoto, Lúcia Rolim Santana de Freitas, Emerson Luiz Lima Araújo, Walter Massa Ramalho

**Affiliations:** 1 Ministério da Saúde, Secretaria de Vigilância em Saúde e Ambiente, Brasília, DF, Brazil; 2 Universidade de Brasília, Faculdade de Medicina, Brasília, DF, Brazil; 3 Ministério da Saúde, Secretaria de Atenção Primária à Saúde, Brasília, DF, Brazil

**Keywords:** Mortality Records, Incidence, Dengue, Public Health Surveillance, Interrupted Time Series Analysis, Registros de Mortalidad, Incidencia, Dengue, Vigilancia de la Salud Pública, Análisis de Series de Tiempo Interrumpidas

## Abstract

**Objective:**

To analyze the temporal trend of dengue incidence and lethality rates and the proportions of its serotypes, in the different macro-regions of Brazil, between 2001 and 2022. In particular, the immediate and gradual effects of these indicators were verified in the periods before and after the publication of the National Guidelines for the Prevention and Control of Dengue Epidemics.

**Methods:**

This was an interrupted time series analysis. Prais-Winsten generalized linear regression was used and the annual percentage variation was determined with a 95% confidence interval (95%CI). The data were extracted from the Notifiable Diseases Information System and the Mortality Information System.

**Results:**

The incidence of dengue in Brazil was stationary from 2001 to 2009, and the effects of the National Guidelines were not detectable between 2010 and 2022. The dengue fatality rate showed an increasing trend in the period 2001-2009. Between 2010 and 2022, there were gradual reductions of 30.0% in the North (95%CI -36.8; -22.5), 27.9% in the Northeast (95%CI -33.3; -22.2), 20.1% in the Southeast (95%CI -30.0; -8.8), and 17.8% in the Center-West (95%CI -22.5; -12.7). For the South, the dengue fatality rate remained stationary between 2001 and 2009 and undetectable between 2010 and 2022.

**Conclusion:**

The trend in the dengue incidence rate in Brazil was stationary in the period 2001-2009. Between 2010 and 2022, it was not possible to detect immediate and gradual effects on incidence rates. The trend in dengue fatality rates in Brazil (except in the South region) was increasing between 2001 and 2009. After 2010, gradual reductions were identified.

## Resumen

**Objetivo:**

Analizar la tendencia temporal de las tasas de incidencia y letalidad del dengue y las proporciones de sus serotipos, en las diferentes macrorregiones de Brasil, entre 2001 y 2022. En particular, se verificaron los efectos inmediatos y graduales de estos indicadores en los períodos anteriores y posteriores a la publicación de las Directrices Nacionales para la Prevención y el Control de las Epidemias de Dengue.

**Métodos:**

Se realizó un análisis de series de tiempo interrumpidas. Se utilizó la regresión lineal generalizada de Prais-Winsten y se determinó la variación porcentual anual con un intervalo de confianza del 95% (IC95%). Los datos fueron extraídos del Sistema de Registro y Notificación de Incidentes y del Sistema de Información de Mortalidad.

**Resultados:**

La incidencia en Brasil fue estacionaria entre 2001 y 2009, y los efectos de las Directrices Nacionales no fueron detectables entre 2010 y 2022. La tasa de letalidad por dengue mostró una tendencia creciente en el período 2001-2009. Entre 2010 y 2022, hubo reducciones graduales del 30,0% en el Norte (IC95% -36,8; -22,5), del 27,9% en el Nordeste (IC95% -33,3; -22,2), del 20,1% en el Sudeste (IC95% -30,0; -8,8) y del 17,8% en el Centro-Oeste (IC95% -22,5; -12,7). En el Sur, la tasa de letalidad por dengue se mantuvo estacionaria entre 2001 y 2009 e indetectable entre 2010 y 2022.

**Conclusión:**

La tendencia de la tasa de incidencia del dengue en Brasil fue estacionaria en el período 2001-2009. Entre 2010 y 2022, no fue posible detectar efectos inmediatos y graduales en las tasas de incidencia. La tendencia de las tasas de mortalidad por dengue en Brasil (excepto en la región Sur) fue creciente entre 2001 y 2009. Después de 2010, se identificaron reducciones graduales.

**Ethical aspects**

This research respected ethical principles, having obtained the following approval data:

Research Ethics Committee: Universidade de Brasília

Opinion number: 6.545.247

Approval date: 30/11/2023

Certificate of Submission for Ethical Appraisal: 75204823.3.0000.5558

Informed Consent Form: Not applicable.

## Introduction

Arboviruses transmitted by *Aedes aegypti* are relevant health problems, especially in countries located in tropical and subtropical regions (1). In 2023, the Americas reported 4,594,823 dengue cases and 2,467 dengue-related deaths (2). In Brazil, in the same year, 1,649,146 probable cases were reported, with an incidence rate of 773/100,000 inhabitants (3).

The current scenario in Brazil is marked by the simultaneous circulation of four dengue virus serotypes, in addition to the Chikungunya and Zika viruses, which makes health surveillance more complex (4). After the creation of the Brazilian National Dengue Control Program in 2002 and the publication of the National Guidelines for the Prevention and Control of Dengue Epidemics in 2009, the country confirmed the introduction of dengue virus serotype 4 in 2010, Chikungunya virus in 2014 and Zika virus in 2015 (5,6).

To reduce the incidence and mortality from vector-borne diseases and prevent epidemics between 2017 and 2030, the World Health Organization and the Brazilian Ministry of Health have proposed response plans (7,8). The control of the *Aedes aegypti* faces challenges related to poor urban infrastructure, low waste collection coverage and intermittent water supply (9-11).

Factors such as rising temperatures and increased human mobility in densely populated areas also contribute to the increased dengue burden (12,13). Since 2016, in response to the public health emergency caused by the Zika virus, the Ministry of Health has invested in research and innovative technologies to improve surveillance and control actions (8,14,15). The introduction of the Wolbachia method and the incorporation of the dengue vaccine into the Unified Health System in 2023 marked a new phase in the fight against the disease (1,15,16).

Interrupted time series analysis, widely used since 2010, allows assessing trends before and after interventions. This quasi-experimental method is ideal for identifying associations between temporal changes and events in health systems, especially when randomization is not possible (17). There are still no studies that use this method to assess the impact of national interventions related to dengue in the last two decades (16-20).

This study analyzed the temporal trends of dengue incidence and lethality rates in the macro-regions of Brazil between 2001 and 2022. The immediate and gradual effects of these indicators were evaluated before and after the publication of the National Guidelines for the Prevention and Control of Dengue Epidemics. The results can guide dengue surveillance strategies in Brazil and other countries in the Americas.

## Methods

### Study design

This study analyzed secondary data from national information systems. Interrupted time series analyses were performed to test the hypothesis that the publication of the National Guidelines for the Prevention and Control of Dengue Epidemics in 2009 caused significant changes in the incidence and lethality rates of the disease’s time series. The objective was to identify changes in the level (abrupt change) or trend (variation in the slope of the time series), using a before-and-after design.

### Context

Brazil is divided into five macro-regions, located in areas of the national territory that reflect their respective names and have specific climatic characteristics. The Southern macro-region has a temperate climate. The North is home to a large part of the Amazon rainforest and has a hot equatorial climate. The Northeast has a tropical climate on the coast and a semi-arid climate in the interior. In the Center-West and Southeast, the tropical climate is predominant.

Probable autochthonous dengue cases were analyzed, by macro-region of residence in Brazil, for the periods 2001-2024, in the exploratory data analysis phase, and 2001-2022, in the interrupted time series analysis. Macro-regions without information on any variable were excluded.

### Variables and measurement

The dependent variables used were the dengue incidence and lethality rates, calculated by macro-regions of Brazil, for the period 2001-2022. The incidence rate was obtained by dividing the number of probable cases by the population of the corresponding year, multiplied by 100,000 inhabitants (2,3,5,6). The years of case reporting were the independent variables.

The fatality rate was calculated by dividing the total number of deaths recorded in the Mortality Information System by the total number of probable dengue cases, multiplied by 100. For each year and macro-region of residence, the total number of deaths by place of residence was used, considering the year of death (selection for row), the macro-region of residence (selection for column) and the International Statistical Classification of Diseases and Related Health Problems, with codes A90 (classical dengue) and A91 (dengue hemorrhagic fever) (21).

Due to underreporting in the Notifiable Diseases Information System (Sinan), which records only a fraction of actual dengue cases (22), it was recommended to calculate incidence and fatality rates based on probable cases, rather than confirmed ones. This practice was supported by publications and manuals from the Ministry of Health (2,3,5,6). To monitor the Sustainable Development Goals, the World Health Organization adopted the same calculation method proposed in this study (7).

The proportion of reported dengue cases by confirmation criteria between 2001 and 2024 and the distribution of predominant serotypes among positive samples were calculated using the molecular polymerase chain reaction technique or viral isolation in the period 2014-2024. These techniques were fundamental for serotype surveillance, as they allowed the identification of changes in viral circulation and were useful in analyzing population risk (23).

### Data sources

Probable cases of dengue, by macro-region of residence and month of onset of symptoms, were obtained from Sinan. The data were available on Tabnet, provided by the Department of Information and Informatics of the Unified Health System (DataSUS), for the periods 2001-2006, 2007-2013 and 2014-2023. The number of deaths between 2001 and 2023 was extracted from the Mortality Information System to calculate the fatality rate (21). 

The resident populations of each macro-region were tabulated based on census data and projections from the Brazilian Institute of Geography and Statistics (IBGE), available on Datasus, for the period 2001-2021 (21). To calculate the 2022 population, IBGE census data were used.

Probable cases and deaths from dengue for 2023 and 2024 were extracted from the Ministry of Health’s Arbovirus Monitoring Panel (until November 1, 2024) (3).

### Participants

The reference population represented the subset of cases reported by healthcare professionals when serving the population that sought public or private health services in Brazil. Since dengue is a disease that requires compulsory notification, each case of a suspected patient treated requires an individual notification form that is completed and entered into Sinan. 

The study population consisted of probable dengue cases reported in Sinan, excluding cases classified as ignored/blank, discarded and inconclusive (21).

### Statistical methods

After tabulating data from 2001 to 2024, trend graphs were created for dengue incidence and lethality rates in Brazil and its macro-regions. These graphs allowed descriptive analysis of the patterns. Graphs with smoothed data were generated using different moving averages.

Based on this analysis and contextual surveys from 2001 to 2024, the main intervention milestones were defined. These milestones were used in the interrupted time series analysis between 2001 and 2022, according to the model best suited to the analytical assumptions.

The assumptions of previous studies on interrupted time series were considered (17,24,25,26). These studies recommended the analysis of at least seven moments before and after the intervention. To assess the trend in dengue incidence and lethality rates before and after the publication of the National Guidelines for the Prevention and Control of Dengue Epidemics in 2009, the interrupted time series method with Prais-Winsten segmented regression was used (27,24).

To calculate the percentages of annual growth or decline, the data were transformed to a logarithmic scale. When the annual percentage change was negative, the trend was decreasing; when it was positive, the trend was increasing. Stationary trends were identified when the 95% confidence intervals included zero or when there was no statistical significance (p-value>0.05). To ensure the absence of serial autocorrelation, the Durbin-Watson statistic (17) was applied.

The hypothesis tested sought to identify changes in the incidence and lethality rates of dengue in Brazil and in its macro-regions before and after the publication of the National Guidelines in 2009. Segmented regression assessed immediate (abrupt change after intervention) and progressive (gradual change) impacts on the temporal trend of these indicators. The post-intervention period between 2010 and 2022 was compared to the pre-intervention period between 2001 and 2009.

Public health authorities developed national guidelines to guide state, municipal and federal managers in the surveillance, prevention, and control of dengue fever. These guidelines aimed to reduce cases and deaths, especially during seasonal or epidemic periods, which contributed to controlling the disease in Brazil (5).

For the exploratory and descriptive analysis, electronic spreadsheets were used. The construction of the figures and the Prais-Winsten segmented regression were performed using R Studio software (version 4.2.0).

## Results

Between 2001 and 2024, 23,454,554 probable cases of dengue were reported in Brazil, with 17,157 deaths, resulting in a fatality rate of 0.07%. The first decade of analysis, between 2001 and 2010, recorded 4,312,491 probable cases, while the second decade, between 2011 and 2020, totaled 9,505,926 cases. In the first five years of the historical series, incidence rates did not exceed 600 cases per 100,000 inhabitants. The incidence rate ranged from 40.5 cases per 100,000 inhabitants in 2004, exceeding 700 cases per 100,000 inhabitants in 2013, 2015, 2016, 2019 and 2024 (Table 1). 

**Table 1:** Number of deaths, probable cases, proportion (%) of dengue cases by laboratory and clinical-epidemiological confirmation criteria, fatality rate (per 100 probable dengue cases) and incidence rate (per 100,000 inhabitants) of dengue by year of notification. Brazil, 2001-2024

Year	Deaths	Probable cases	Fatality rate	Proportion (%) of cases by clinical-epidemiological confirmation criterion	Proportion (%) of cases by laboratory confirmation criteria	Incidence rate	Total probable cases in the period
2001	42	389,324	0.01	40.6	31.9	225.8	4,312,491^a^
2002	166	700,583	0.02	52.8	17.9	401.1	
2003	69	277,421	0.02	50.4	30.3	156.8	
2004	26	72,552	0.04	48.8	38.1	40.5	
2005	48	151,413	0.03	47.9	36.7	82.2	
2006	114	263,890	0.04	40.8	42.5	141.2	
2007	332	501,745	0.07	36.0	35.4	265.0	
2008	589	558,023	0.11	43.1	21.3	294.3	
2009	375	416,264	0.09	50.1	27.4	217.3	
2010	774	981,276	0.08	51.3	33.5	514.4	
2011	604	686,005	0.09	50.8	33.5	356.5	9,505,926^b^
2012	390	580,519	0.07	41.5	24.7	299.2	
2013	642	1,428,989	0.04	50.9	29.6	710.8	
2014	458	591,549	0.08	45.6	38.9	291.7	
2015	930	1,709,159	0.05	49.4	31.4	835.9	
2016	853	1,497,956	0.06	51.0	21.5	726.8	
2017	210	241,884	0.09	50.0	17.8	116.4	
2018	239	269.301	0.09	49.7	23.6	128.7	
2019	902	1,553,579	0.06	56.6	27.2	737.4	
2020	685	946,985	0.07	49.0	33.4	446.5	
2021	404	544,812	0.07	36.6	46.8	255.2	9,636,137^c^
2022	1,279	1,243,487	0.10	47.3	41.7	612.3	
2023^d^	1.306	1,297,064	0.10	44.7	41.0	599.7	
2024^d^	5,720	6,550,774	0.09	54.1	32.0	3,226.0	

^a^Cases for the period 2001-2010; ^b^Cases for the period 2011-2020; ^c^Cases for the period 2021-2024; ^d^Data subject to review.

**Figure 1:**
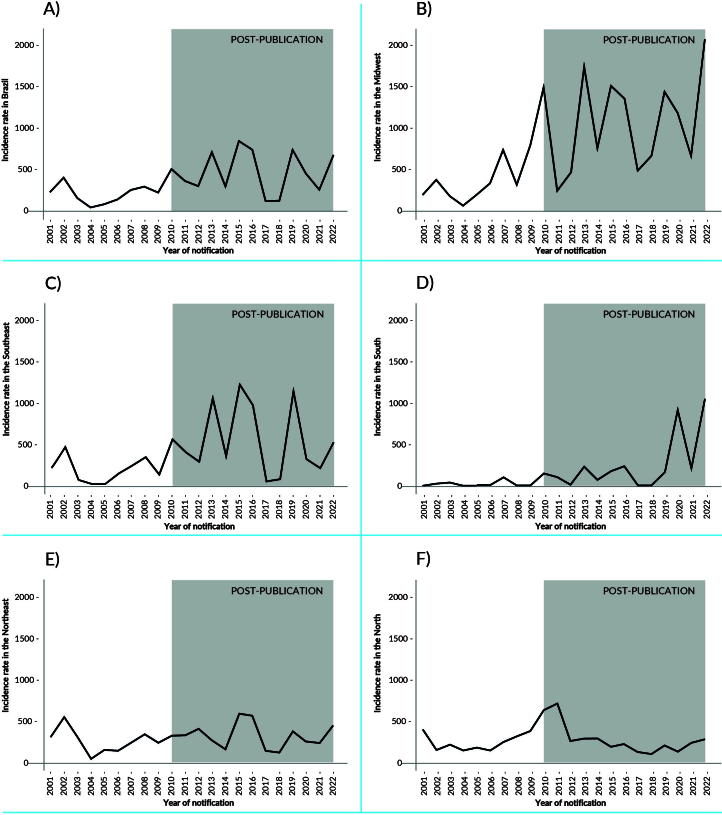
Dengue incidence rate (per 100,000 inhabitants) before and after the publication of the National Guidelines for the Prevention and Control of Dengue Epidemics, according to the year of notification for Brazil (A) and the Central-West (B), Southeast (C), South (D), Northeast (E) and North (F) macro-regions. Brazil and macro-regions, 2001-2022

**Figure 2:**
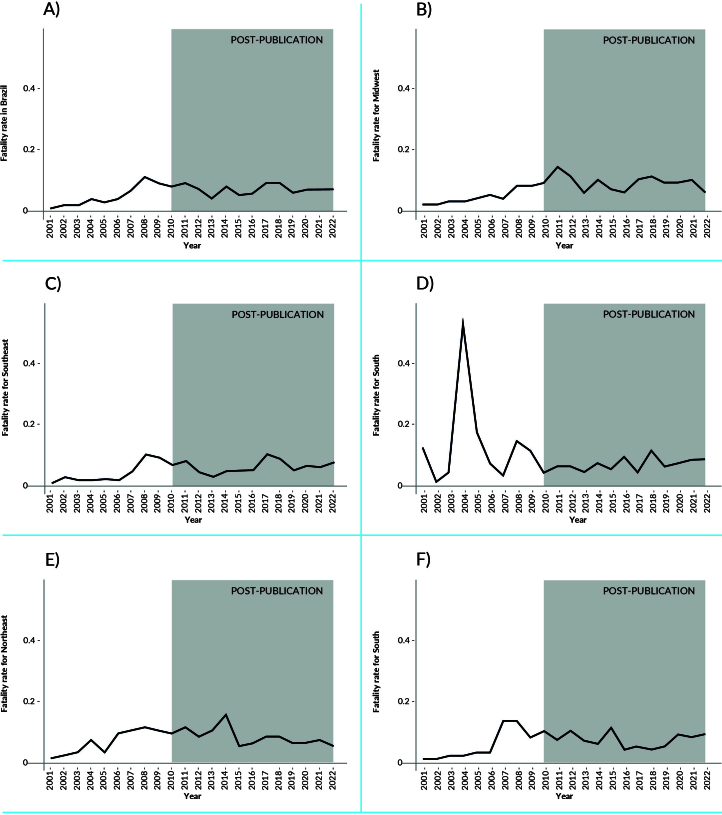
Dengue fatality rate (per 100 probable dengue cases), before and after the publication of the National Guidelines for the Prevention and Control of Dengue Epidemics, according to death at residence for Brazil (A) and the Central-West (B), Southeast (C), South (D), Northeast (E) and North (F) macro-regions. Brazil and macro-regions, 2001-2022

At the beginning of the historical series, the Southern macro-region had the lowest dengue incidence rate. In 2020, this rate in the Southern macro-region was 921 cases per 100,000 inhabitants, followed by a reduction in 2021 and a significant increase in 2022 (Figure 1). From 2009 onwards, high incidence rates were observed in the Central-West macro-region, with 823 cases per 100,000 inhabitants in 2009, 1,505 cases per 100,000 inhabitants in 2010, 1,747 cases in 2013 and 1,447 cases per 100,000 inhabitants in 2019 (Figure 1; Supplementary Table 1). The highest fatality rate in the country was identified in the Southern macro-region at the beginning of the series (Figure 2).

To identify the temporal trend of dengue incidence and lethality rates in Brazil and its macro-regions from 2001 to 2022, and the effect of interventions in the Unified Health System from 2009 onwards, segmented regression analysis was performed for interrupted time series.

The trend in the dengue incidence rate for Brazil and its macro-regions was stationary in the period 2001-2009 with annual percentage variation of 1.5% (95%CI -16.7; 23.7; p-value 0.882). Interrupted time series analysis revealed no significant effects for Brazil or any macro-region in the period 2010-2022 (Table 2). 

**Table 2:** Annual percentage variation (APV) and 95% confidence interval (95%CI) of dengue incidence rates (per 100,000 inhabitants). Brazil and macro-regions, 2001-2022

Dimension	Regressors	Interpretation	APV (95%CI)	p-value	Durbin-Watson test^a^
Brazil	Change of level (step)	Not detected	131.6 (-35.8;736.8)	0.216	1.80
	Trend change (ramp)	Not detected	-2.2 (-22.5;23.5)	0.856	
	Time	Stationary	1.5 (-16.7;23.7)	0.882	
North	Change of level (step)	Not detected	77.6 (-24.7;319.2)	0.206	1.93
	Trend change (ramp)	Not detected	-9.0 (-23.3;7.9)	0.292	
	Time	Stationary	1.4 (-11.6;16.5)	0.842	
Northeast	Change of level (step)	Not detected	66.4 (-45.3;406.9)	0.382	1.86
	Trend change (ramp)	Not detected	2.0 (-16.4;24.6)	0.847	
	Time	Stationary	-3.1 (-18.3;14.8)	0.718	
South	Change of level (step)	Not detected	456.8 (-63.9;8,503.6)	0.235	1.90
	Trend change (ramp)	Not detected	14.9 (-29.3;86.9)	0.582	
	Time	Stationary	-1.8 (-35.3;49.0)	0.931	
Southeast	Change of level (step)	Not detected	292.6 (-38.0;2,389.3)	0.164	1.81
	Trend change (ramp)	Not detected	-5.4 (-32.5;32.5)	0.749	
	Time	Stationary	1.1 (-23.9;34.5)	0.939	
Center-West	Change of level (step)	Not detected	21.9 (-56.9;245.3)	0.714	1.99
	Trend change (ramp)	Not detected	-10.7 (-25.5;6.9)	0.232	
	Time	Stationary	17.5 (0.3;37.5)	0.060	

^a^A value close to or equal to 2 means the absence of serial autocorrelation.

Interrupted time series analysis for Brazil showed an immediate reduction of 36.7% (95%CI -54.0; -12.7; p-value 0.012) and a progressive reduction of 22.0% (95%CI -26.1; -17.6; p-value 0.002) in the fatality rate, in relation to the underlying increasing trend of 28.6% (95%CI 22.5; 34.9; p-value<0.001), after the publication of the National Guidelines for the Prevention and Control of Dengue Epidemics (Table 3).

**Table 3:** Annual percentage variation (APV) and 95% confidence interval (95%CI) of dengue fatality rate (per 100 probable dengue cases). Brazil and macro-regions, 2001-2022

Dimension	Regressors	Interpretation	APV (95%CI)	p-value	Durbin Watson Test^a^
Brazil	Time	Growing	28.6 (22.5;34.9)	<0.001	2.08
	Change of level (step)	Abrupt reduction	-36.7 (-54.0;-12.7)	0.012	
	Trend change (ramp)	Progressive reduction	-22.0 (-26.1;-17.6)	0.002	
North	Time	Growing	40.8 (28.7;53.9)	<0.001	1.94
	Change of level (step)	Not detected	-41.5 (-67.6;-5.4)	0.059	
	Trend change (ramp)	Progressive reduction	-30.0 (-36.8;-22.5)	<0.001	
Northeast	Time	Growing	32.2 (23.5;41.6)	0.003	2.04
	Change of level (step)	Not detected	-27.7 (-54.0;)	0.176	
	Trend change (ramp)	Progressive reduction	-27.9 (-33.3;-22.2)	0.002	
South	Time	Stationary	9.1 (-9.2;31.3)	0.364	2.03
	Change of level (step)	Not detected	-59.3 (-88.0;37.8)	0.166	
	Trend change (ramp)	Not detected	-4.2 (-22.4;18.2)	0.696	
Southeast	Time	Growing	28.3 (14.8;43.4)	<0.001	1.86
	Change of level (step)	Not detected	-37.1 (-69.2;28.4)	0.219	
	Trend change (ramp)	Progressive reduction	-20.1 (-30.0;-8.8)	0.004	
Center-West	Time	Growing	19.7 (13.6;26.1)	0.005	2.01
	Change of level (step)	Not detected	25.4 (-11.2;77.2)	0.215	
	Trend change (ramp)	Progressive reduction	-17.8 (-22.5;-12.7)	0.007	

^a^A value close to or equal to 2 means the absence of serial autocorrelation.

The fatality rate showed an increasing trend between 2001 and 2009, but there was a change in trend with gradual reductions (ramp) in the period 2010-2022. Reductions were 30.0% in the North (95%CI -36.8; -22.5; p-value<0.001), 27.9% in the Northeast (95%CI -33.3; -22.2; p-value 0.002), 20.1% in the Southeast (95%CI -30.0; -8.8; p-value 0.004) and 17.8% in the Central-West (95%CI -22.5; -12.7; p-value 0.007). In the Southern macro-region, the fatality rate remained stationary between 2001 and 2009 (95%CI -9.2; 31.3; p-value 0.364) and did not show significant variations (not detected) between 2010 and 2022, either in level or slope (Table 3). 

The confirmation of dengue cases by clinical-epidemiological criteria predominated throughout the historical series in Brazil. The highest proportions of cases confirmed by laboratory criteria were 46.8% in 2021, 42.5% in 2006 and 41.7% in 2022 (Table 1).

The four serotypes of the dengue virus circulated in Brazil, with changes in predominance over the years. Between 2014 and 2023, serotypes 1 and 2 were predominant. Serotype 1 was identified in more than 60.0% of positive samples with the molecular technique polymerase chain reaction or viral isolation from 2021 onwards, remaining predominant in the Southeast, Northeast, Central-West and South macro-regions. In 2023, serotype 2 predominated in the North, while serotype 3 was identified in 4.0% of positive samples in this macro-region (Supplementary Table 2).

## Discussion

Between 2001 and 2024, there was an increase in reported cases of dengue fever and in the fatality rate in Brazil, especially after the COVID-19 pandemic. At the beginning of the historical series, the South macro-region had the lowest incidence rate, but, in more recent years, it stood out with the highest rate, followed by the Center-West. The increase in morbidity and mortality due to dengue in Brazil and worldwide has been associated with urbanization, climate change and greater human mobility (13,28-30). Social, environmental, and economic aspects favor the spread of the vector, while host characteristics influence the severity of the disease (1,9,13,23,30).

Following the Zika epidemic in 2016, the incidence of dengue and Zika decreased in 2017 and 2018 and re-emerged in 2019. This reduction may be related to herd immunity or the circulation of dengue virus strains at low levels until local conditions favor new outbreaks (31,32).

The results reinforce the importance of the National Guidelines for the Prevention and Control of Dengue Epidemics, especially in *Aedes aegypti* surveillance actions. Although the interrupted time series analysis did not identify effects of the guidelines on incidence rates, there was an ecological effect on fatality rates for all macro-regions, except the South. Before the intervention, between 2001 and 2009, the trend was increasing, but after 2010 there were gradual reductions.

The high mortality rate from dengue fever directly reflects the quality of health care (29,33). The decline in case fatality rate between 2010 and 2022 suggested that national guidelines may have contributed more significantly to patient care than to vector control.

Investments in social determinants and innovative technologies, such as the dengue vaccine and the Wolbachia method, can positively impact the incidence of dengue (15,23). The expansion of dengue fever to the South (16,34), previously less affected, signaled the influence of climate change and reinforced the need for professional qualifications to identify and clinically manage serious cases.

 Dengue virus serotype 2 was associated with dengue severity in children (35). There are many gaps in the molecular epidemiology of dengue (36), and it is necessary to better study the predominance of serotype 2 in recent years and in different age groups. Dengue virus serotype 2 was introduced in 1990 in Southeast Brazil. Serotypes 1 and 4 were introduced into Brazil, from the Northern macro-region, in 1981 (37). The introduction of serotype 3 into Brazilian territory was confirmed, from the Northern macro-region, in 1999 (37). Although serotype 3 was identified in 4.0% of dengue-positive samples in 2023 in the North, two decades later, the same serotype returned in 2024 and was reported in different macro-regions of Brazil (3,16).

The results of the distribution of different dengue serological tests, with the change in the predominant serotype over the years and the simultaneous circulation of other viruses or arboviruses, have raised the discussion related to the theory of competition between ecological niches and zoonotic diseases (38,39). These changes have reinforced the need for continuous laboratory surveillance, especially through molecular methods, such as the polymerase chain reaction or viral isolation, which help in understanding the transmission dynamics and in the timely identification of changes in the serological profile (23,35,37). 

The response to the COVID-19 pandemic, from 2020 onwards, expanded molecular diagnostic capacity globally (40). The increase in case confirmations using laboratory criteria in 2020 and 2021 could be associated with the strengthening of the public health laboratory network of the Unified Health System. 

Limitations of this study included possible underreporting in Sinan and in deaths recorded in the Mortality Information System. Such problems may underestimate or overestimate incidence and fatality rates, which affects the accuracy of analyses (22). Data up to 2022 may be outdated due to changes in health systems during the COVID-19 pandemic (18).

Laboratory surveillance, particularly molecular identification, plays a vital role in understanding dengue dynamics (23,35,37). Strengthening this surveillance, combined with improving the completeness and consistency of data in Sinan, is essential for robust epidemiological analyses. Ensuring accurate records allow for better monitoring of the spread of the disease and planning of effective interventions.

For future interrupted time series analyses, new milestones should be considered, given the scenario of implementation of cost-effective technologies in controlling *Aedes aegypti* in Brazil. With the start of dengue vaccination for children and adolescents, it is crucial to discuss expanding vaccination coverage to other age groups.

The interrupted time series analysis method is a valuable tool for evaluating the effectiveness of the vaccine and other surveillance, prevention, and control strategies, and should be recommended and developed by managers and professionals from different areas of activity in the Unified Health System.

In conclusion, dengue remains a growing challenge in Brazil. Interrupted time series analysis did not detect any effects of national guidelines on incidence rates but showed a positive impact on reducing fatality rates after 2010. This finding highlights the need to improve surveillance actions, with a focus on incorporating innovative technologies and work processes, aiming to reduce morbidity and mortality and improve the health system’s response.

## Data Availability

The database and analysis codes used in the research are available at: https://github.com/tatianeportal/base-interrupted-time-series-analysis-dengue

## Supplementary material

Supplementary Table 1 and Supplementary Table 2.
