# Supplementary material for: Trends in dengue incidence and lethality: interrupted time series analysis, Brazil, 2001-2022
Source: Epidemiol Serv Saude. 2025 Sep 8;34:e20240424. doi: 10.1590/S2237-96222025v34e20240424.en (PMC12435890; doi:10.1590/S2237-96222025v34e20240424.en)
Supplement: Supplementary file 1 [file 2237-9622-ress-34-e20240424-suppl01-en.pdf]

Supplementary Table 1. Proportion (%) of dengue cases confirmed by confirmation criteria, according to year of notification. Brazil and macro-regions, 2001-2023

**Region**	**2001**	**2002**	**2003**	**2004**	**2005**	**2006**	**2007**	**2008**	**2009**	**2010**	**2011**	**2012**	**2013**	**2014**	**2015**	**2016**	**2017**	**2018**	**2019**	**2020**	**2021**	**2022**	**2023**
Ignored or blank	27.6	29.3	19.2	13.1	15.4	16.7	28.5	35.6	22.5	15.2	15.1	31.6	18.5	14.5	17.5	26.3	29.8	23.6	14.7	16.3	15.2	10.4	25.3
Laboratory	31.9	17.9	30.3	38.1	36.7	42.5	35.4	21.3	27.4	33.5	33.5	24.7	29.6	38.9	31.4	21.5	17.8	23.6	27.2	33.4	46.8	41.7	35.8
Clinical-epidemiological	40.6	52.8	50.4	48.8	47.9	40.8	36.0	43.1	50.1	51.3	50.8	41.5	50.9	45.6	49.4	51.0	50,0	49.7	56.6	49.0	36.6	47.3	36.2
Under investigation	0.0	0.0	0.0	0.0	0.0	0.0	0.0	0.0	0.0	0.0	0.6	2.2	1.0	1.0	1.7	1,2	2.3	3.1	1.6	1,2	1.4	0.7	2.7

Supplementary Table 2. Proportion (%) of reported cases according to predominant dengue serotypes among samples positive by molecular technique polymerase chain reaction or viral isolation. Brazil and macro-regions, 2014-2023

Macro-region	**Serotypes**	**2014**	**2015**	**2016**	**2017**	**2018**	**2019**	**2020**	**2021**	**2022**	**2023**
Northeast	1	201/553(36.3)	653/698 (93.6)	169/193 (87.6)	39/49 (79.6)	134/153 (87.6)	971/1,249 (77.7)	265/358 (74.0)	105/495 (21.2)	858/1.986 (43.2)	795/1,284 (61.9)
	2	7/553 (1.3)	10/698 (1.4)	5/193 (2.6)	6/49 (12.2)	16/153 (10.5)	271/1,249 (21.7)	88/358 (24.6)	386/495 (78.0)	1,120/1,986 (56.4)	486/1,284 (37.9)
	3	8/553 (1.4)	13/698 (1.9)	3/193 (1.6)	0/49 (0.0)	2/153 (1.3)	2/1,249 (0.2)	0/358 (0.0)	3/495 (0.6)	3/1.986 (0.2)	1/1,284 (0.1)
	4	337/553 (60.9)	22/698 (3.2)	16/193 (8.3)	4/49 (8.2)	1/153 (0.7)	5/1,249 (0.4)	5/358 (1.4)	1/495 (0.2)	5/1,986 (0.3)	2/1,284 (0.2)
North	1	68/141 (48.2)	297/343 (86.6)	86/120 (71.7)	95/104 (91.3)	53/203 (26.1)	172/514 (33.5)	104/204 (51.0)	918/1,027 (89.4)	1,629/1,780 (91.5)	941/2,007 (46.9)
	2	3/141 (2.1)	5/343 (1.5)	22/120 (18.3)	6/104 (5.8)	148/203 (72.9)	341/514 (66.3)	98/204 (48.0)	107/1,027 (10.4)	150/1,780 (8.4)	985/2,007 (49.1)
	3	2/141 (1.4)	1/343 (0.3)	3/120 (2.5)	1/104 (1.0)	2/203 (1.0)	0/514 (0.0)	0/204 (0.0)	0/1,027 (0.0)	0/1,780 (0.0)	77/2,007 (3.8)
	4	68/141 (48.2)	40/343 (11.7)	9/120 (7.5)	2/104 (1.9)	0/203 (0.0)	1/514 (0.2)	2/204 (1.0)	2/1,027 (0.2)	1/1,780 (0.1)	4/2,007 (0.2)
Center-West	1	413/510 (81.0)	1,290/1,423 (90.7)	541/616 (87.8)	79/265 (29.8)	65/271 (24.0)	434/1,981 (21.9)	699/1,956 (35.7)	475/881 (53.9)	4,531/4,567 (99.2)	6,991/7,172 (97.5)
	2	6/510 (1.2)	29/1,423 (2.0)	39/616 (6.3)	175/265 (66.0)	198/271 (73.1)	1,527/1,981 (77.1)	1,248/1,956 (63.8)	399/881 (45.3)	28/4,567 (0.6)	178/7,172 (2.5)
	3	0/510 (0.0)	4/1,423 (0.3)	5/616 (0.8)	1/265 (0.4)	0/271 (0.0)	1/1,981 (0.1)	0/1,956 (0.0)	0/881 (0.0)	0/4,567 (0.0)	1/7,172 (0.0)

Macro-region	**Serotypes**	**2014**	**2015**	**2016**	**2017**	**2018**	**2019**	**2020**	**2021**	**2022**	**2023**
	4	91/510 (17.8)	100/1,423 (7.0)	31/616 (5.0)	10/265 (3.8)	8/271 (3.0)	19/1,981 (1.0)	9/1,956 (0.5)	7/881 (0.8)	8/4,567 (0.2)	2/7,172 (0.0)
South	1	259/267 (97.0)	452/473 (95.6)	1.944/1,984 (98.0)	8/10 (80.0)	54/99 (54.5)	2,247/5,896 (38.1)	651/3,077 (21.2)	722/1,053 (68.6)	7,916/8,412 (94.1)	8,560/8,675 (98.7)
	2	2/267 (0.7)	2/473 (0.4)	7/1,984 (0.4)	1/10 (10.0)	44/99 (44.4)	3,293/5,896 (55.9)	2,405/3,077 (78.2)	331/1,053 (31.4)	491/8,412 (5.8)	108/8,675 (1.2)
	3	0/267 (0.0)	0/473 (0.0)	27/1,984 (1.4)	0/10 (0.0)	0/99 (0.0)	0/5,896 (0.0)	2/3,077 (0.1)	0/1,053 (0.0)	1/8,412 (0.0)	1/8,675 (0.0)
	4	6/267 (2.2)	19/473 (4.0)	6/1,984 (0.3)	1/10 (10.0)	1/99 (1.0)	356/5,896 (6.0)	19/3,077 (0.6)	0/1,053 (0.0)	4/8,412 (0.0)	6/8,675 (0.1)
Southeast	1	1,397/1,519 (92.0)	2,521/2,732 (92.3)	1,010/1,119 (90.3)	60/112 (53.6)	148/482 (30.7)	399/2,988 (13.4)	326/1,530 (21.3)	522/858 (60.8)	3,245/3,929 (82.6)	(10,843/12,009 (90.3)
	2	13/1,519 (0.9)	29/2,732 (1.1)	57/1,119 (5.1)	17/112 (15.2)	329/482 (68.3)	2,569/2,988 (86.0)	1,200/1,530 (78.4)	336/858 (39.2)	683/3,929 (17.4)	1,158/12,009 (9.6)
	3	1/1,519 (0.1)	6/2.732 (0.2)	3/1,119 (0.3)	7/112 (6.3)	1/482 (0.2)	5/2,988 (0.2)	1/1,530 (0.1)	0/858 (0.0)	0/3,929 (0.0)	1/12,009 (0.0)
	4	108/1,519 (7.1)	176/2,732 (6.4)	49/1,119 (4.4)	28/112 (25.0)	4/482 (0.8)	15/2,988 (0.5)	3/1,530 (0.2)	0/858 (0.0)	1/3,929 (0.0)	7/12,009 (0.1)
Brazil	1	2,339/2,991 (78.2)	5,216/5,673 (91.9)	3,752/4,034 (93.0)	281/540 (52.0)	456/1,210 (37.7)	4,223/12,628 (33.4)	2,045/7,125 (28.7)	2,744/4,316 (63.6)	18,183/20,679 (87.9)	27,771/30,267 (91.8)
	2	31/2,991 (1.0)	75/5,673 (1.3)	130/4,034 (3.2)	205/540 (38.0)	735/1,210 (60.7)	8,001/12,628 (63.4)	5,039/7,125 (70.7)	1559/4,316 (36.1)	2473/20,676 (12.0)	2,465/30,267 (8.1)
	3	11/2,991 (0.4)	24/5,673 (0.4)	41/4,034 (1.0)	9/540 (1.7)	5/1,210 (0.4)	8/12,628 (0.1)	3/7,125 (0.0)	3/4,316 (0.1)	4/20,676 (0.0)	12/30,267 (0.0)
	4	610/2,991 (20.4)	358/5,673 (6.3)	111/4,034 (2.8)	45/540 (8.3)	14/1,210(1.2)	396/12,628 (3.1)	38/7,125 (0.5)	10/4,316 (0.2)	19/20,676 (0.1)	19/30,267 (0.1)
