# Supplementary material for: Trends in dengue incidence and lethality: interrupted time series analysis, Brazil, 2001-2022
Source: Epidemiol Serv Saude. 2025 Sep 8;34:e20240424. doi: 10.1590/S2237-96222025v34e20240424.en (PMC12435890; doi:10.1590/S2237-96222025v34e20240424.en)
Supplement: Supplementary file 2 [file 2237-9622-ress-34-e20240424-suppl01-pt.pdf]

Tabela suplementar 1. Proporção (%) dos casos de dengue confirmados por critério de confirmação, segundo ano de notificação. Brasil e macrorregiões, 2001-2023

**Região**	**2001**	**2002**	**2003**	**2004**	**2005**	**2006**	**2007**	**2008**	**2009**	**2010**	**2011**	**2012**	**2013**	**2014**	**2015**	**2016**	**2017**	**2018**	**2019**	**2020**	**2021**	**2022**	**2023**
Ignorado ou em branco	27,6	29,3	19,2	13,1	15,4	16,7	28,5	35,6	22,5	15,2	15,1	31,6	18,5	14,5	17,5	26,3	29,8	23,6	14,7	16,3	15,2	10,4	25,3
Laboratorial	31,9	17,9	30,3	38,1	36,7	42,5	35,4	21,3	27,4	33,5	33,5	24,7	29,6	38,9	31,4	21,5	17,8	23,6	27,2	33,4	46,8	41,7	35,8
Clínico-epidemiológico	40,6	52,8	50,4	48,8	47,9	40,8	36,0	43,1	50,1	51,3	50,8	41,5	50,9	45,6	49,4	51,0	50,0	49,7	56,6	49,0	36,6	47,3	36,2
Em investigação	0,0	0,0	0,0	0,0	0,0	0,0	0,0	0,0	0,0	0,0	0,6	2,2	1,0	1,0	1,7	1,2	2,3	3,1	1,6	1,2	1,4	0,7	2,7

Tabela suplementar 2. Proporção (%) dos casos notificados segundo sorotipos predominantes de dengue entre as amostras positivas por técnica molecular reação em cadeia da polimerase ou isolamento viral. Brasil e macrorregiões, 2014-2023

**Macrorregião**	**Sorotipos**	**2014**	**2015**	**2016**	**2017**	**2018**	**2019**	**2020**	**2021**	**2022**	**2023**
Nordeste	1	201/553(36,3)	653/698 (93,6)	169/193 (87,6)	39/49 (79,6)	134/153 (87,6)	971/1.249 (77,7)	265/358 (74,0)	105/495 (21,2)	858/1.986 (43,2)	795/1.284 (61,9)
	2	7/553 (1,3)	10/698 (1,4)	5/193 (2,6)	6/49 (12,2)	16/153 (10,5)	271/1.249 (21,7)	88/358 (24,6)	386/495 (78,0)	1.120/1.986 (56,4)	486/1.284 (37,9)
	3	8/553 (1,4)	13/698 (1,9)	3/193 (1,6)	0/49 (0,0)	2/153 (1,3)	2/1.249 (0,2)	0/358 (0,0)	3/495 (0,6)	3/1.986 (0,2)	1/1.284 (0,1)
	4	337/553 (60,9)	22/698 (3,2)	16/193 (8,3)	4/49 (8,2)	1/153 (0,7)	5/1.249 (0,4)	5/358 (1,4)	1/495 (0,2)	5/1.986 (0,3)	2/1.284 (0,2)
Norte	1	68/141 (48,2)	297/343 (86,6)	86/120 (71,7)	95/104 (91,3)	53/203 (26,1)	172/514 (33,5)	104/204 (51,0)	918/1.027 (89,4)	1.629/1.780 (91,5)	941/2.007 (46,9)
	2	3/141 (2,1)	5/343 (1,5)	22/120 (18,3)	6/104 (5,8)	148/203 (72,9)	341/514 (66,3)	98/204 (48,0)	107/1.027 (10,4)	150/1.780 (8,4)	985/2.007 (49,1)
	3	2/141 (1,4)	1/343 (0,3)	3/120 (2,5)	1/104 (1,0)	2/203 (1,0)	0/514 (0,0)	0/204 (0,0)	0/1.027 (0,0)	0/1.780 (0,0)	77/2.007 (3,8)
	4	68/141 (48,2)	40/343 (11,7)	9/120 (7,5)	2/104 (1,9)	0/203 (0,0)	1/514 (0,2)	2/204 (1,0)	2/1.027 (0,2)	1/1.780 (0,1)	4/2.007 (0,2)
Centro-Oeste	1	413/510 (81,0)	1.290/1.423 (90,7)	541/616 (87,8)	79/265 (29,8)	65/271 (24,0)	434/1.981 (21,9)	699/1.956 (35,7)	475/881 (53,9)	4.531/4.567 (99,2)	6.991/7.172 (97,5)
	2	6/510 (1,2)	29/1.423 (2,0)	39/616 (6,3)	175/265 (66,0)	198/271 (73,1)	1.527/1.981 (77,1)	1.248/1.956 (63,8)	399/881 (45,3)	28/4.567 (0,6)	178/7.172 (2,5)
	3	0/510 (0,0)	4/1.423 (0,3)	5/616 (0,8)	1/265 (0,4)	0/271 (0,0)	1/1.981 (0,1)	0/1.956 (0,0)	0/881 (0,0)	0/4.567 (0,0)	1/7.172 (0,0)
	4	91/510 (17,8)	100/1.423 (7,0)	31/616 (5,0)	10/265 (3,8)	8/271 (3,0)	19/1.981 (1,0)	9/1.956 (0,5)	7/881 (0,8)	8/4.567 (0,2)	2/7.172 (0,0)

Macrorregião	Sorotipos	2014	2015	2016	2017	2018	2019	2020	2021	2022	2023
Sul	1	259/267 (97,0)	452/473 (95,6)	1.944/1.984 (98,0)	8/10 (80,0)	54/99 (54,5)	2.247/5.896 (38,1)	651/3.077 (21,2)	722/1.053 (68,6)	7.916/8.412 (94,1)	8.560/8.675 (98,7)
	2	2/267 (0,7)	2/473 (0,4)	7/1.984 (0,4)	1/10 (10,0)	44/99 (44,4)	3.293/5.896 (55,9)	2.405/3.077 (78,2)	331/1.053 (31,4)	491/8.412 (5,8)	108/8.675 (1,2)
	3	0/267 (0,0)	0/473 (0,0)	27/1.984 (1,4)	0/10 (0,0)	0/99 (0,0)	0/5.896 (0,0)	2/3.077 (0,1)	0/1.053 (0,0)	1/8.412 (0,0)	1/8.675 (0,0)
	4	6/267 (2,2)	19/473 (4,0)	6/1.984 (0,3)	1/10 (10,0)	1/99 (1,0)	356/5.896 (6,0)	19/3.077 (0,6)	0/1.053 (0,0)	4/8.412 (0,0)	6/8.675 (0,1)
Sudeste	1	1.397/1.519 (92,0)	2.521/2.732 (92,3)	1.010/1.119 (90,3)	60/112 (53,6)	148/482 (30,7)	399/2.988 (13,4)	326/1.530 (21,3)	522/858 (60,8)	3.245/3.929 (82,6)	(10.843/12.009 (90,3)
	2	13/1.519 (0,9)	29/2.732 (1,1)	57/1.119 (5,1)	17/112 (15,2)	329/482 (68,3)	2.569/2.988 (86,0)	1.200/1.530 (78,4)	336/858 (39,2)	683/3.929 (17,4)	1.158/12.009 (9,6)
	3	1/1.519 (0,1)	6/2.732 (0,2)	3/1.119 (0,3)	7/112 (6,3)	1/482 (0,2)	5/2.988 (0,2)	1/1.530 (0,1)	0/858 (0,0)	0/3.929 (0,0)	1/12.009 (0,0)
	4	108/1.519 (7,1)	176/2.732 (6,4)	49/1.119 (4,4)	28/112 (25,0)	4/482 (0,8)	15/2.988 (0,5)	3/1.530 (0,2)	0/858 (0,0)	1/3.929 (0,0)	7/12.009 (0,1)
Brasil	1	2.339/2.991 (78,2)	5.216/5.673 (91,9)	3.752/4.034 (93,0)	281/540 (52,0)	456/1.210 (37,7)	4.223/12.628 (33,4)	2.045/7.125 (28,7)	2.744/4.316 (63,6)	18.183/20.679 (87,9)	27.771/30.267 (91,8)
	2	31/2.991 (1,0)	75/5.673 (1,3)	130/4.034 (3,2)	205/540 (38,0)	735/1.210 (60,7)	8.001/12.628 (63,4)	5.039/7.125 (70,7)	1559/4.316 (36,1)	2473/20.676 (12,0)	2.465//30.267 (8,1)
	3	11/2.991 (0,4)	24/5.673 (0,4)	41/4.034 (1,0)	9/540 (1,7)	5/1.210 (0,4)	8/12.628 (0,1)	3/7.125 (0,0)	3/4.316 (0,1)	4/20.676 (0,0)	12//30.267 (0,0)
	4	610/2.991 (20,4)	358/5.673 (6,3)	111/4.034 (2,8)	45/540 (8,3)	14/1.210(1,2)	396/12.628 (3,1)	38/7.125 (0,5)	10/4.316 (0,2)	19/20.676 (0,1)	19//30.267 (0,1)
