# Supplementary material for: Trends in dengue incidence and lethality: interrupted time series analysis, Brazil, 2001-2022
Source: Epidemiol Serv Saude. 2025 Sep 8;34:e20240424. doi: 10.1590/S2237-96222025v34e20240424.en (PMC12435890; doi:10.1590/S2237-96222025v34e20240424.en)
Supplement: Translation

# Translation: Tendências na incidência e letalidade da dengue: análise de séries temporais interrompida, Brasil, 2001-2022

**DOI:** 10.1590/S2237-96222025v34e20240424.pt

**Authors:** Tatiane Fernandes Portal de Lima Alves da Silva, Henry Maia Peixoto, Lúcia Rolim Santana de Freitas, Emerson Luiz Lima Araújo, Walter Massa Ramalho

**Affiliations:** 1 Ministério da Saúde, Secretaria de Vigilância em Saúde e Ambiente, Brasília, DF, Brasil; 2 Universidade de Brasília, Faculdade de Medicina, Brasília, DF, Brasil; 3 Ministério da Saúde, Secretaria de Atenção Primária à Saúde, Brasília, DF, Brasil

**Keywords:** Registros de Mortalidade, Incidência, Dengue, Vigilância em Saúde Pública, Análise de Séries Temporais Interrompida

## Abstract

**Objetivo:**

Analisar a tendência temporal das taxas de incidência e de letalidade da dengue e as proporções dos seus sorotipos, nas diversas macrorregiões do Brasil, entre 2001 e 2022. Foram verificados, notadamente, os efeitos imediatos e graduais desses indicadores nos períodos antes e após a publicação das Diretrizes Nacionais para Prevenção e Controle de Epidemias da Dengue.

**Métodos:**

Tratou-se de análise de séries temporais interrompida. Utilizou-se regressão linear generalizada de Prais-Winsten e foi determinada a variação percentual anual com intervalo de confiança de 95% (IC95%). Os dados foram extraídos do Sistema de Informação de Agravos de Notificação e do Sistema de Informação sobre Mortalidade.

**Resultados:**

A incidência no Brasil foi estacionária de 2001 a 2009, e os efeitos das Diretrizes Nacionais não foram detectáveis entre 2010 e 2022. A taxa de letalidade da dengue apresentou tendência crescente no período 2001-2009. Entre 2010 e 2022, houve reduções graduais de 30,0% no Norte (IC95% -36,8; -22,5), 27,9% no Nordeste (IC95% -33,3; -22,2), 20,1% no Sudeste (IC95% -30,0; -8,8) e 17,8% no Centro-Oeste (IC95% -22,5; -12,7). Para o Sul, a taxa de letalidade da dengue manteve-se estacionária entre 2001 e 2009 e não detectável entre 2010 e 2022.

**Conclusão:**

A tendência da taxa de incidência de dengue no Brasil foi estacionária no período 2001-2009. Entre 2010 e 2022, não foi possível detectar efeitos imediatos e graduais nas taxas de incidência. A tendência das taxas de letalidade da dengue no Brasil (exceto na região Sul) foi crescente entre 2001 e 2009. Após 2010, foram identificadas reduções graduais.

**Aspectos éticos**

A presente pesquisa respeitou os princípios éticos, obtendo os seguintes dados de aprovação:

Comitê de ética em pesquisa: Universidade de Brasília

Número do parecer: 6.545.247

Data de aprovação: 30/11/2023

Certificado de apresentação de apreciação ética: 75204823.3.0000.5558

Registro do consentimento livre e esclarecido: Não se aplica.

## Introdução

As arboviroses transmitidas pelo *Aedes aegypti* são relevantes problemas de saúde, principalmente em países situados nas regiões tropicais e subtropicais (1). Em 2023, as Américas reportaram 4.594.823 casos de dengue e 2.467 mortes relacionadas à doença (2). No Brasil, no mesmo ano, foram notificados 1.649.146 casos prováveis, com taxa de incidência de 773/100 mil habitantes (3).

O cenário atual no Brasil é marcado pela circulação simultânea dos quatro sorotipos do vírus da dengue, além dos vírus Chikungunya e Zika, o que torna a vigilância em saúde mais complexa (4). Após a criação do Programa Nacional de Controle da Dengue em 2002 e a publicação das Diretrizes Nacionais para Prevenção e Controle de Epidemias da Dengue em 2009, o país confirmou a introdução do sorotipo 4 do vírus da dengue em 2010, do vírus Chikungunya em 2014 e do vírus Zika em 2015 (5,6).

Para reduzir a incidência e a mortalidade por doenças transmitidas por vetores e prevenir epidemias entre 2017 e 2030, a Organização Mundial da Saúde e o Ministério da Saúde do Brasil propuseram planos de enfrentamento (7,8). O controle do *Aedes aegypti* enfrenta desafios relacionados à infraestrutura urbana precária, à baixa cobertura de coleta de lixo e ao abastecimento de água intermitente (9-11).

Fatores como o aumento das temperaturas e a intensificação da mobilidade humana em áreas densamente povoadas também contribuem para o aumento da carga da dengue (12,13). Desde 2016, em resposta à emergência de saúde pública causada pelo vírus Zika, o Ministério da Saúde tem investido em pesquisas e novas tecnologias para aprimorar as ações de vigilância e controle (8,14,15). A introdução do método Wolbachia e a incorporação da vacina contra a dengue no Sistema Único de Saúde em 2023 marcaram uma nova fase no combate à doença (1,15,16).

A análise de séries temporais interrompida, amplamente utilizada desde 2010, permite avaliar tendências antes e depois de intervenções. Esse método quase experimental é ideal para identificar associações entre mudanças temporais e eventos em sistemas de saúde, especialmente quando a randomização não é possível (17). Ainda não há estudos que utilizem esse método para avaliar o impacto de intervenções nacionais relacionadas à dengue nas últimas duas décadas (16-20).

Este estudo analisou as tendências temporais das taxas de incidência e letalidade da dengue, nas macrorregiões do Brasil, entre 2001 e 2022. Avaliaram-se os efeitos imediatos e graduais desses indicadores antes e após a publicação das Diretrizes Nacionais para Prevenção e Controle de Epidemias da Dengue. Os resultados podem orientar estratégias de vigilância da dengue no Brasil e em outros países das Américas.

## Métodos

### Delineamento

Este estudo analisou dados secundários de sistemas nacionais de informações. Análises de séries temporais interrompida foram realizadas para testar a hipótese de que a publicação das Diretrizes Nacionais para Prevenção e Controle de Epidemias de Dengue, em 2009, provocou mudanças significativas nas taxas de incidência e letalidade das séries temporais da doença. O objetivo foi identificar alterações no nível (mudança abrupta) ou na tendência (variação na inclinação da série temporal), utilizando o delineamento do tipo antes e depois.

### Contexto

O Brasil é dividido em cinco macrorregiões, localizadas em áreas do território nacional que refletem os respectivos nomes e possuem características climáticas específicas. A macrorregião Sul apresenta clima temperado. O Norte abriga grande parte da floresta amazônica e tem clima equatorial quente. O Nordeste tem clima tropical no litoral e semiárido no interior. No Centro-Oeste e no Sudeste, o clima tropical é predominante.

Analisaram-se os casos prováveis de dengue autóctones, por macrorregião de residência no Brasil, para os períodos 2001-2024, na fase de análise exploratória dos dados, e 2001-2022, na análise de séries temporais interrompida. Macrorregiões sem informações em qualquer variável foram excluídas.

### Variáveis e mensuração

As variáveis dependentes utilizadas foram as taxas de incidência e de letalidade da dengue, calculadas por macrorregiões do Brasil, para o período 2001-2022. A taxa de incidência foi obtida dividindo-se o número de casos prováveis pela população do ano correspondente, multiplicado por 100 mil habitantes (2,3,5,6). Os anos de notificação dos casos foram as variáveis independentes.

A taxa de letalidade foi calculada dividindo-se o número total de óbitos registrados no Sistema de Informação sobre Mortalidade pelo número total de casos prováveis de dengue, multiplicado por 100. Para cada ano e macrorregião de residência, utilizou-se o total de óbitos por local de residência, considerando o ano do óbito (seleção para linha), a macrorregião de residência (seleção para coluna) e a Classificação Estatística Internacional de Doenças e Problemas Relacionados à Saúde, com os códigos A90 (dengue clássica) e A91 (febre hemorrágica da dengue) (21).

Devido à subnotificação no Sistema de Informação de Agravos de Notificação (Sinan), que registra apenas uma fração dos casos reais de dengue (22), recomendou-se calcular as taxas de incidência e letalidade com base nos casos prováveis, em vez dos confirmados. Essa prática foi respaldada por publicações e manuais do Ministério da Saúde (2,3,5,6). Para monitorar os Objetivos de Desenvolvimento Sustentável, a Organização Mundial da Saúde adotou o mesmo método de cálculo proposto neste estudo (7).

Foram calculadas a proporção de casos notificados de dengue por critério de confirmação entre 2001 e 2024 e a distribuição dos sorotipos predominantes entre as amostras positivas, por técnica molecular de reação em cadeia da polimerase ou isolamento viral no período 2014-2024. Essas técnicas foram fundamentais para a vigilância de sorotipos, pois permitiram identificar mudanças na circulação viral e foram úteis na análise de risco populacional (23).

### Fontes de dados

Os casos prováveis de dengue, por macrorregião de residência e mês de início dos sintomas, foram obtidos do Sinan. Os dados estavam disponíveis no Tabnet, fornecido pelo Departamento de Informação e Informática do Sistema Único de Saúde (DataSUS), para os períodos 2001-2006, 2007-2013 e 2014-2023. O número de óbitos entre 2001 e 2023 foi extraído do Sistema de Informação sobre Mortalidade para o cálculo da taxa de letalidade (21). 

As populações residentes de cada macrorregião foram tabuladas com base nos dados censitários e nas projeções do Instituto Brasileiro de Geografia e Estatística (IBGE), disponíveis no Datasus, para o período 2001-2021 (21). Para o cálculo da população de 2022, foram utilizados dados censitários do IBGE.

Os casos prováveis e os óbitos por dengue referentes a 2023 e 2024 foram extraídos do Painel de Monitoramento das Arboviroses do Ministério da Saúde (até 1º de novembro de 2024) (3).

### Participantes

A população de referência representou o subconjunto dos casos notificados pelos profissionais de saúde ao atender a população que buscou os serviços de saúde públicos ou privados no Brasil. Sendo a dengue uma doença de notificação compulsória, cada caso de paciente com suspeita atendido deveria gerar uma ficha individual de notificação preenchida e inserida no Sinan. 

A população de estudo foi a dos casos prováveis de dengue notificados no Sinan, excluídos os casos classificados como ignorados/branco, descartados e inconclusivos (21).

### Métodos estatísticos

Após a tabulação dos dados de 2001 a 2024, foram construídos gráficos de tendência para as taxas de incidência e letalidade da dengue no Brasil e suas macrorregiões. Esses gráficos permitiram a análise descritiva dos padrões. Gráficos com dados suavizados foram gerados, utilizando diferentes médias móveis.

Com base nessa análise e em levantamentos contextuais de 2001 a 2024, definiram-se os principais marcos de intervenção. Esses marcos foram utilizados na análise de séries temporais interrompida entre 2001 e 2022, conforme o modelo mais adequado aos pressupostos analíticos.

Foram considerados os pressupostos de estudos prévios sobre séries temporais interrompidas (17,24,25,26). Esses estudos recomendaram a análise de, pelo menos, sete momentos antes e depois da intervenção. Para avaliar a tendência das taxas de incidência e letalidade da dengue antes e após a publicação, em 2009, das Diretrizes Nacionais para Prevenção e Controle de Epidemias de Dengue, utilizou-se o método de séries temporais interrompida com regressão segmentada de Prais-Winsten (27,24).

Para calcular os percentuais de crescimento ou declínio anual, os dados foram transformados em escala logarítmica. Quando a variação percentual anual foi negativa, a tendência foi decrescente; quando positiva, a tendência foi crescente. Tendências estacionárias foram identificadas quando os intervalos de confiança de 95% incluíam zero ou quando não havia significância estatística (p-valor>0,05). Para garantir a ausência de autocorrelação serial, aplicou-se a estatística de Durbin-Watson (17).

A hipótese testada buscou identificar mudanças nas taxas de incidência e letalidade da dengue no Brasil e em suas macrorregiões antes e após a publicação das Diretrizes Nacionais em 2009. A regressão segmentada avaliou impactos imediatos (mudança abrupta após a intervenção) e progressivos (mudança gradual) na tendência temporal desses indicadores. O período pós-intervenção entre 2010 e 2022 foi comparado ao período pré-intervenção entre 2001 e 2009.

As diretrizes nacionais foram elaboradas por autoridades de saúde pública para orientar gestores estaduais, municipais e federais na vigilância, na prevenção e no controle da dengue. Essas orientações visaram reduzir casos e óbitos, especialmente durante períodos sazonais ou epidêmicos, o que contribuiu para o controle da doença no Brasil (5).

Para a análise exploratória e descritiva, utilizaram-se planilhas eletrônicas. A construção das figuras e a regressão segmentada de Prais-Winsten foram realizadas no software R Studio (versão 4.2.0).

## Resultados

Entre 2001 e 2024, foram notificados 23.454.554 casos prováveis de dengue no Brasil, com 17.157 óbitos, o que resultou na taxa de letalidade de 0,07%. A primeira década de análise, entre 2001 e 2010, registrou 4.312.491 casos prováveis, enquanto a segunda década, entre 2011 e 2020, somou 9.505.926 casos. No primeiro quinquênio da série histórica, as taxas de incidência não ultrapassavam 600 casos por 100 mil habitantes. A taxa de incidência variou de 40,5 casos por 100 mil habitantes em 2004, ultrapassando 700 casos por 100 mil habitantes em 2013, 2015, 2016, 2019 e 2024 (Tabela 1). 

**Tabela 1:** Número de óbitos, casos prováveis, proporção (%) de casos de dengue por critério de confirmação laboratorial e clínico-epidemiológico, taxa de letalidade (por 100 casos prováveis) e taxa de incidência (por 100 mil habitantes) de dengue por ano de notificação. Brasil, 2001-2024

Ano	Óbitos	Casos prováveis	Taxa de letalidade	Proporção (%) de casos por critério de confirmação clínico-epidemiológico	Proporção (%) de casos por critério de confirmação laboratorial	Taxa de incidência	Total de casos prováveis no período
2001	42	389.324	0,01	40,6	31,9	225,8	4.312.491^a^
2002	166	700.583	0,02	52,8	17,9	401,1	
2003	69	277.421	0,02	50,4	30,3	156,8	
2004	26	72.552	0,04	48,8	38,1	40,5	
2005	48	151.413	0,03	47,9	36,7	82,2	
2006	114	263.890	0,04	40,8	42,5	141,2	
2007	332	501.745	0,07	36,0	35,4	265,0	
2008	589	558.023	0,11	43,1	21,3	294,3	
2009	375	416.264	0,09	50,1	27,4	217,3	
2010	774	981.276	0,08	51,3	33,5	514,4	
2011	604	686.005	0,09	50,8	33,5	356,5	9.505.926^b^
2012	390	580.519	0,07	41,5	24,7	299,2	
2013	642	1.428.989	0,04	50,9	29,6	710,8	
2014	458	591.549	0,08	45,6	38,9	291,7	
2015	930	1.709.159	0,05	49,4	31,4	835,9	
2016	853	1.497.956	0,06	51,0	21,5	726,8	
2017	210	241.884	0,09	50,0	17,8	116,4	
2018	239	269.301	0,09	49,7	23,6	128,7	
2019	902	1.553.579	0,06	56,6	27,2	737,4	
2020	685	946.985	0,07	49,0	33,4	446,5	
2021	404	544.812	0,07	36,6	46,8	255,2	9.636.137^c^
2022	1.279	1.243.487	0,10	47,3	41,7	612,3	
2023^d^	1.306	1.297.064	0,10	44,7	41,0	599,7	
2024^d^	5.720	6.550.774	0,09	54,1	32,0	3.226,0	

^a^Casos para o período 2001-2010; ^b^Casos para o período 2011-2020; ^c^Casos para o período 2021-2024; ^d^Dados sujeitos à revisão.

No início da série histórica, a macrorregião Sul apresentou a menor taxa de incidência de dengue. Em 2020, essa taxa na macrorregião Sul foi de 921 casos por 100 mil habitantes, seguida por redução em 2021 e aumento expressivo em 2022 (Figura 1). A partir de 2009, observaram-se altas taxas de incidência na macrorregião Centro-Oeste, com 823 casos por 100 mil habitantes em 2009, 1.505 casos por 100 mil habitantes em 2010, 1.747 casos em 2013 e 1.447 casos por 100 mil habitantes em 2019 (Figura 1; Tabela suplementar 1). A maior taxa de letalidade do país foi identificada na macrorregião Sul no início da série (Figura 2).

**Figura 2:** Taxa de letalidade (por 100 casos prováveis), antes e após a publicação das Diretrizes Nacionais para Prevenção e Controle de Epidemias de Dengue, segundo óbito de residência para o Brasil (A) e macrorregiões Centro-Oeste (B), Sudeste (C), Sul (D), Nordeste (E) e Norte (F). Brasil e macrorregiões, 2001-2022

**Figura 1:** Taxa de incidência de dengue (por 100 mil habitantes) antes e após a publicação das *Diretrizes Nacionais para Prevenção e Controle de Epidemias de Dengue*, segundo ano de notificação para o Brasil (A) e macrorregiões Centro-Oeste (B), Sudeste (C), Sul (D), Nordeste (E) e Norte (F). Brasil e macrorregiões, 2001-2022

Para identificar a tendência temporal das taxas de incidência e letalidade da dengue no Brasil e nas macrorregiões de 2001 a 2022, e o efeito das intervenções no Sistema Único de Saúde a partir de 2009, foi realizada análise de regressão segmentada para séries temporais interrompida.

A tendência da taxa de incidência de dengue para o Brasil e suas macrorregiões foi estacionária no período 2001-2009 com variação percentual anual de 1,5% (IC95% -16,7; 23,7; p-valor 0,882). A análise de séries temporais interrompida não revelou efeitos significativos para o Brasil ou para qualquer macrorregião no período 2010-2022 (Tabela 2). 

**Tabela 2:** Variação percentual anual (VPA) e intervalo de confiança de 95% (IC95%) das taxas de incidência da dengue (por 100 mil habitantes). Brasil e macrorregiões, 2001-2022

Dimensão	Regressores	Interpretação	VPA (IC95%)	p-valor	Teste Durbin-Watson^a^
Brasil	Mudança de nível (degrau)	Não detectada	131,6 (-35,8; 736,8)	0,216	1,80
	Mudança de tendência (rampa)	Não detectada	-2,2 (-22,5; 23,5)	0,856	
	Tempo	Estacionário	1,5 (-16,7; 23,7)	0,882	
Norte	Mudança de nível (degrau)	Não detectada	77,6 (-24,7; 319,2)	0,206	1,93
	Mudança de tendência (rampa)	Não detectada	-9,0 (-23,3; 7,9)	0,292	
	Tempo	Estacionário	1,4 (-11,6; 16,5)	0,842	
Nordeste	Mudança de nível (degrau)	Não detectada	66,4 (-45,3; 406,9)	0,382	1,86
	Mudança de tendência (rampa)	Não detectada	2,0 (-16,4; 24,6)	0,847	
	Tempo	Estacionário	-3,1 (-18,3; 14,8)	0,718	
Sul	Mudança de nível (degrau)	Não detectada	456,8 (-63,9; 8.503,6)	0,235	1,90
	Mudança de tendência (rampa)	Não detectada	14,9 (-29,3; 86,9)	0,582	
	Tempo	Estacionário	-1,8 (-35,3; 49,0)	0,931	
Sudeste	Mudança de nível (degrau)	Não detectada	292,6 (-38,0; 2.389,3)	0,164	1,81
	Mudança de tendência (rampa)	Não detectada	-5,4 (-32,5; 32,5)	0,749	
	Tempo	Estacionário	1,1 (-23,9; 34,5)	0,939	
Centro-Oeste	Mudança de nível (degrau)	Não detectada	21,9 (-56,9; 245,3)	0,714	1,99
	Mudança de tendência (rampa)	Não detectada	-10,7 (-25,5; 6,9)	0,232	
	Tempo	Estacionário	17,5 (0,3; 37,5)	0,060	

^a^Valor próximo ou igual a 2 significa ausência de autocorrelação serial.

A análise de séries temporais interrompida para o Brasil mostrou redução imediata de 36,7% (IC95% -54,0; -12,7; p-valor 0,012) e redução progressiva de 22,0% (IC95% -26,1; -17,6; p-valor 0,002) na taxa de letalidade, em relação à tendência subjacente crescente de 28,6% (IC95% 22,5; 34,9; p-valor<0,001), após a publicação das Diretrizes Nacionais para Prevenção e Controle de Epidemias da Dengue (Tabela 3).

**Tabela 3:** Variação percentual anual (VPA) e intervalo de confiança de 95% (IC95%) das taxas de letalidade da dengue (por 100 casos de dengue). Brasil e macrorregiões, 2001-2022

Dimensão	Regressores	Interpretação	VPA (IC95%)	p-valor	Teste Durbin Watson^a^
Brasil	Tempo	Crescente	28,6 (22,5; 34,9)	<0,001	2,08
	Mudança de nível (degrau)	Redução abrupta	-36,7 (-54,0; -12,7)	0,012	
	Mudança de tendência (rampa)	Redução progressiva	-22,0 (-26,1; -17,6)	0,002	
Norte	Tempo	Crescente	40,8 (28,7; 53,9)	<0,001	1,94
	Mudança de nível (degrau)	Não detectada	-41,5 (-67,6; -5,4)	0,059	
	Mudança de tendência (rampa)	Redução progressiva	-30,0 (-36,8; -22,5)	<0,001	
Nordeste	Tempo	Crescente	32,2 (23,5; 41,6)	0,003	2,04
	Mudança de nível (degrau)	Não detectada	-27,7 (-54,0; 13,6)	0,176	
	Mudança de tendência (rampa)	Redução progressiva	-27,9 (-33,3; -22,2)	0,002	
Sul	Tempo	estacionário	9,1 (-9,2; 31,3)	0,364	2,03
	Mudança de nível (degrau)	Não detectada	-59,3 (-88,0; 37,8)	0,166	
	Mudança de tendência (rampa)	Não detectada	-4,2 (-22,4; 18,2)	0,696	
Sudeste	Tempo	Crescente	28,3 (14,8; 43,4)	<0,001	1,86
	Mudança de nível (degrau)	Não detectada	-37,1 (-69,2; 28,4)	0,219	
	Mudança de tendência (rampa)	Redução progressiva	-20,1 (-30,0; -8,8)	0,004	
Centro-Oeste	Tempo	crescente	19,7 (13,6; 26,1)	0,005	2,01
	Mudança de nível (degrau)	Não detectada	25,4 (-11,2; 77,2)	0,215	
	Mudança de tendência (rampa)	Redução progressiva	-17,8 (-22,5; -12,7)	0,007	

^a^Valor próximo ou igual a 2 significa ausência de autocorrelação serial.

A taxa de letalidade apresentou tendência crescente entre 2001 e 2009, mas houve mudança de tendência com reduções graduais (em rampa) no período 2010-2022. As reduções foram de 30,0% no Norte (IC95% -36,8; -22,5; p-valor<0,001), 27,9% no Nordeste (IC95% -33,3; -22,2; p-valor 0,002), 20,1% no Sudeste (IC95% -30,0; -8,8; p-valor 0,004) e 17,8% no Centro-Oeste (IC95% -22,5; -12,7; p-valor 0,007). Na macrorregião Sul, a taxa de letalidade manteve-se estacionária entre 2001 e 2009 (IC95% -9,2; 31,3; p-valor 0,364) e não apresentou variações significativas (não detectada) entre 2010 e 2022, tanto em nível quanto em rampa (Tabela 3). 

A confirmação de casos de dengue pelo critério clínico-epidemiológico predominou ao longo da série histórica no Brasil. As maiores proporções de casos confirmados por critério laboratorial foram 46,8% em 2021, 42,5% em 2006 e 41,7% em 2022 (Tabela 1).

Os quatro sorotipos do vírus da dengue circularam no Brasil, com mudanças na predominância ao longo dos anos. Entre 2014 e 2023, os sorotipos 1 e 2 foram predominantes. O sorotipo 1 foi identificado em mais de 60,0% das amostras positivas da técnica molecular reação em cadeia da polimerase ou isolamento viral a partir de 2021, mantendo-se predominante nas macrorregiões Sudeste, Nordeste, Centro-Oeste e Sul. Em 2023, o sorotipo 2 predominou no Norte, enquanto o sorotipo 3 foi identificado em 4,0% das amostras positivas nessa macrorregião (Tabela suplementar 2).

## Discussão

Entre 2001 e 2024, observou-se o aumento nos casos notificados de dengue no Brasil e na taxa de letalidade, especialmente após a pandemia de covid-19. No início da série histórica, a macrorregião Sul apresentou a menor taxa de incidência, mas, nos anos mais recentes, destacou-se com a maior taxa, seguida pelo Centro-Oeste. O aumento da morbimortalidade por dengue no Brasil e no mundo tem sido associado à urbanização, às mudanças climáticas e à maior mobilidade humana (13,28-30). Aspectos sociais, ambientais e econômicos favorecem a propagação do vetor, enquanto características do hospedeiro influenciam a gravidade da doença (1,9,13,23,30).

Após a epidemia de zika em 2016, a incidência de dengue e zika diminuiu em 2017 e 2018 e ressurgiu em 2019. Essa redução pode estar relacionada à imunidade coletiva ou à circulação de linhagens do vírus da dengue em baixos níveis até que condições locais favoreçam novos surtos (31,32).

Os resultados reforçam a importância das Diretrizes Nacionais para Prevenção e Controle de Epidemias de Dengue, especialmente nas ações de vigilância do *Aedes aegypti*. Embora a análise de séries temporais interrompida não tenha identificado efeitos das diretrizes nas taxas de incidência, houve o efeito ecológico nas taxas de letalidade para todas as macrorregiões, exceto o Sul. Antes da intervenção, entre 2001 e 2009, a tendência era de aumento, mas após 2010 houve reduções graduais.

A elevada letalidade por dengue reflete diretamente a qualidade da assistência em saúde (29,33). O declínio na taxa de letalidade entre 2010 e 2022 sugeriu que as diretrizes nacionais podem ter contribuído mais significativamente para a assistência aos pacientes do que para o controle do vetor.

Investimentos em determinantes sociais e novas tecnologias, como a vacina contra a dengue e o método Wolbachia, podem impactar positivamente a incidência da dengue (15,23). A expansão da dengue para o Sul (16,34), antes menos afetado, sinalizou a influência das mudanças climáticas e reforçou a necessidade de qualificação profissional para identificação e manejo clínico de casos graves.

O sorotipo 2 do vírus Dengue associou-se à gravidade da dengue em crianças (35). Há muitas lacunas na epidemiologia molecular na dengue (36), sendo necessário estudar melhor o predomínio do sorotipo 2 em anos recentes e em diferentes faixas etárias. O sorotipo 2 do vírus da Dengue foi introduzido em 1990 no Sudeste brasileiro. Os sorotipos 1 e 4 foram introduzidos no Brasil, a partir da macrorregião Norte, em 1981 (37). A introdução do sorotipo 3 no território brasileiro foi confirmada, a partir da macrorregião Norte, em 1999 (37). Apesar de o sorotipo 3 ter sido identificado em 4,0% nas amostras positivas para dengue em 2023 no Norte, duas décadas seguintes, o mesmo sorotipo voltou a circular em 2024 e foi reportado em distintas macrorregiões do Brasil (3,16).

Os resultados da distribuição dos diferentes sorológicos da dengue, com a alteração do sorotipo predominante ao longo dos anos e a circulação simultânea de outros vírus ou arbovírus, suscitaram a discussão relacionada à teoria de competição de nichos ecológicos e doenças zoonóticas (38,39). Essas alterações reforçaram a necessidade de vigilância laboratorial contínua, especialmente por meio de métodos moleculares, como técnica molecular reação em cadeia da polimerase ou isolamento viral, que auxiliam na compreensão da dinâmica de transmissão e na identificação oportuna de mudanças no perfil sorológico (23,35,37). 

A resposta à pandemia de covid-19, a partir de 2020, ampliou a capacidade de diagnóstico molecular nos países (40). O aumento das confirmações dos casos pelo critério laboratorial em 2020 e 2021 puderam estar associadas ao fortalecimento da rede de laboratórios de saúde pública do Sistema Único de Saúde. 

As limitações deste estudo incluíram possíveis subnotificações no Sinan e nos óbitos registrados no Sistema de Informações sobre Mortalidade. Tais problemas podem subestimar ou superestimar as taxas de incidência e letalidade, o que afeta a precisão das análises (22). Os dados até 2022 podem estar defasados devido às mudanças nos sistemas de saúde durante a pandemia de covid-19 (18).

A vigilância laboratorial, particularmente a identificação molecular, desempenha papel central na compreensão da dinâmica da dengue (23,35,37). O fortalecimento dessa vigilância, aliado à melhoria na completude e consistência dos dados no Sinan, é essencial para análises epidemiológicas robustas. Garantir registros precisos permite monitorar melhor a propagação da doença e planejar intervenções efetivas.

Para futuras análises de séries temporais interrompida, novos marcos devem ser considerados, dado o cenário de implementação de tecnologias custo-efetivas no controle do *Aedes aegypti* no Brasil. Com o início da vacinação contra a dengue para crianças e adolescentes, é crucial discutir a ampliação da cobertura vacinal para outras faixas etárias.

O método de análise de séries temporais interrompida é ferramenta valiosa para avaliar a efetividade da vacina e de outras estratégias de vigilância, prevenção e controle, devendo ser recomendado e desenvolvido pelos gestores e profissionais de diferentes instâncias de atuação no Sistema Único de Saúde.

Conclui-se que a dengue permanece como desafio crescente no Brasil. A análise de séries temporais interrompida não detectou efeitos das diretrizes nacionais nas taxas de incidência, mas evidenciou impacto positivo na redução das taxas de letalidade após 2010. Esse achado ressalta a necessidade de aprimorar as ações de vigilância, com foco na incorporação de novas tecnologias e processos de trabalho, visando reduzir morbimortalidade e aprimorar a resposta do sistema de saúde.

## Data Availability

O banco de dados e os códigos de análise utilizados na pesquisa estão disponíveis em: https://github.com/tatianeportal/base-interrupted-time-series-analysis-dengue

## Material suplementar

Tabela suplementar 1 e Tabela suplementar 2.
